# Premature Coronary Artery Disease and Familial Dyslipidemia in Patients Presenting With Acute Coronary Syndrome: A Tertiary Cardiac Center Registry

**DOI:** 10.7759/cureus.103992

**Published:** 2026-02-20

**Authors:** Mohamed Saad, Mohammad Alshehri, Amr Elgazzar, Mohamed Senara, Hesham S Taha

**Affiliations:** 1 Cardiology, Prince Khalid Bin Sultan Cardiac Center, Armed Forces Hospitals Southern Region, Khamis Muchait, SAU

**Keywords:** acute coronary syndrome, dyslipidemia, familial hypercholestrolemia, lipid profiles, premature coronary artery disease

## Abstract

Background: Premature coronary artery disease (CAD) in patients presenting with acute coronary syndrome (ACS) is an increasing clinical concern driven by genetic predisposition and lifestyle factors. This study compared clinical and laboratory characteristics of premature versus non-premature CAD and identified predictors of early-onset disease.

Methods: This was a cross-sectional observational study of 2,000 patients admitted with confirmed ACS. Premature CAD was defined as males <55 years and females <65 years. Clinical, laboratory, and cardiological variables were compared between groups.

Results: From December 2021 to March 2025, 2,000 patients were enrolled. Premature CAD occurred in 637 patients (31.9%), who were younger (median age 49 years) and predominantly male (68.4%). Smoking (32.8% vs. 9.4%), family history of dyslipidemia (20.4% vs. 3.7%), higher total cholesterol (174.0 vs. 145.0 mg/dL, p<.001), higher low-density lipoprotein (LDL) cholesterol (108.3 vs. 85.1 mg/dL, p<.001), and higher corrected LDL (155.8 vs. 147.3 mg/dL, p<.001) were more common in premature CAD, whereas diabetes and hypertension were less frequent. Independent predictors included smoking (OR 4.71, 95% CI 3.68 6.03), family history of dyslipidemia (OR 6.73, 95% CI 4.78 9.48), cholesterol ≥200 mg/dL (OR 2.26, 95% CI 1.80 2.83), LDL ≥100 mg/dL (OR 2.10, 95% CI 1.74 2.54), and hypertriglyceridemia (OR 1.77, 95% CI 1.45 2.17).

Conclusions: Premature CAD in patients presenting with ACS demonstrates a risk profile dominated by smoking and familial dyslipidemia rather than metabolic comorbidities. Early lipid screening and aggressive modification of lifestyle risk factors are essential to reduce early atherosclerotic disease burden.

## Introduction

Premature coronary artery disease (CAD) refers to the development of CAD as acute coronary syndrome (ACS) at a younger age, typically before 55 years in men and 65 years in females [1]. One of the most prominent contributors to premature CAD is familial hypercholesterolemia (FH), a genetic condition marked by persistently elevated low-density lipoprotein cholesterol (LDL-C) levels from childhood, which significantly raises the risk of early-onset atherosclerotic cardiovascular disease (ASCVD) [2,3]. Although FH affects nearly one in every 250 individuals globally, it often goes undetected and untreated, especially in resource-limited settings [4]. The constant exposure to high LDL-C in FH accelerates endothelial damage and atherosclerotic plaque formation, frequently leading to ACS in younger individuals [5]. In many cases, an ACS event may be the first clinical indication of underlying FH, highlighting the need for timely identification and intervention [6]. In this context, it becomes crucial to define the clinical and biochemical features of premature CAD more precisely, particularly in patients presenting with ACS given that these individuals are often at heightened but underrecognized risk.

Exploring the patterns and prevalence of dyslipidemia, including FH and other hereditary or metabolic factors, is vital for improving screening protocols, enhancing risk prediction, and informing early preventive strategies. Moreover, social determinants of health such as socioeconomic status, education level, access to healthcare, and urban lifestyle also contribute significantly to the development and outcomes of premature CAD. These upstream factors can influence both the onset and management of premature CAD and should be considered when designing effective public health interventions [7].

This study aims to investigate the relationship between FH and premature CAD in patients admitted with ACS. Through the analysis of clinical profiles, lipid data, FH diagnostic scores, and procedural details, the research seeks to delineate the unique risk factors of premature CAD and reinforce the necessity of prompt diagnosis and aggressive lipid-lowering interventions in high-risk younger adults. In addition to genetic and lipid-related risk factors, unhealthy dietary habits, increased consumption of saturated fats and refined carbohydrates, and elevated body mass index (BMI) are recognized contributors to early atherosclerosis and should be considered in comprehensive preventive strategies even though these variables were not captured in our dataset.

## Materials and methods

Study design

A cross-sectional observational study was conducted at the Department of Cardiology, Prince Khaled Bin Sultan Hospital, between December 2021 and March 2025. The study population consisted of 2,000 adult patients consecutively admitted with a confirmed diagnosis of ACS. Participants were classified into two groups based on age and sex: premature CAD (males under 55 years and females under 65 years) and non-premature CAD (males 55 years or older and females 65 years or older).

Study procedures

Data was obtained from electronic hospital records and included demographics, cardiovascular risk factors, laboratory test results, echocardiographic and angiographic findings, and details of in-hospital treatment. Lipid measurements were collected within 24 hours of admission. FH was identified using both the Dutch Lipid Clinic Network (DLCN) and Simon Broome criteria. Diagnosis of ACS was established based on internationally recognized guidelines [8,9]. Clinical features suggestive of dyslipidemia or FH, such as tendon xanthomas and corneal arcus, were evaluated [10]. LDL-C was assessed using a direct detect spectrometer (Abbott Alinity C system; Abbott Park, IL, USA). For patients already on lipid-lowering therapy at the time of admission, LDL-C was retrospectively estimated using correction factors derived from published medication efficacy data [11-15]. In-hospital interventions including medication use, reperfusion strategies, and discharge prescriptions were recorded. To convert lipid values into mg/dL, the multiplication factors used were 38.67 for cholesterol (total, high-density lipoprotein (HDL), LDL-C) and 88.57 for triglycerides.

Inclusion criteria

Patients aged 18 years or older were eligible if they had a confirmed diagnosis of ACS based on one or more of the following: clinical presentation, electrocardiogram findings, elevated cardiac biomarkers, or coronary angiography demonstrating ≥50% luminal narrowing. Only those with unstable angina, non-ST-elevation myocardial infarction (NSTEMI), or ST-elevation myocardial infarction (STEMI) and complete datasets were included.

Exclusion criteria

Exclusion criteria included incomplete lipid profiles, blood samples collected beyond 24 hours of Coronary Care Unit (CCU) admission, triglyceride levels >400 mg/dL, hemodynamic instability, cardiogenic shock, chronic kidney disease (estimated glomerular filtration rate (eGFR) < 60 mL/min/1.73 m²), acute renal failure at admission, or alcohol consumption exceeding 14 units per week.

Statistical analysis

All patient data were anonymized before analysis. Statistical analyses were carried out using IBM SPSS Statistics for Windows, Version 27.0 (IBM Corp., Armonk, NY, USA). Categorical data were expressed as frequencies and percentages and compared using Pearson's chi-square, Fisher's exact, or Fisher-Freeman-Halton tests, as appropriate. Continuous variables were assessed for normality. Normally distributed data were reported as mean ± standard deviation (SD); skewed variables were presented as median and interquartile range (IQR). Comparisons between groups used the Mann-Whitney U test. Logistic regression was employed to identify factors associated with premature CAD, with odds ratios (ORs) and 95% confidence intervals (CIs) reported. A p-value <.05 was considered statistically significant.

## Results

Among 2,000 ACS patients, 637 (31.9%) had premature CAD and 1,363 (68.1%) had non-premature CAD. Patients with premature CAD were younger (median age 49 [IQR 44-54] vs. 71 [IQR 65-79], p<0.001) and more frequently male (68.4%). Smoking was more prevalent (32.8% vs. 9.4%, p<0.001), as was a family history of dyslipidemia (20.4% vs. 3.7%, p<0.001). in contrast, diabetes (52.7% vs 72.9%; p<0.001), hypertension (53.7% vs. 76.0%, p<0.001) and prior myocardial infarction (11.6% vs. 16.4%, p=0.005) were less frequent in the premature CAD group, as shown in Table 1. cardiological assessment revealed that ST segment elevation was more common in premature CAD (29.4% vs. 23.0%, p=0.002).

**Table 1:** Patient characteristics. Data are presented as counts (percentages) or medians (interquartile range). Age ‘71 (65, 79)’ and ‘49 (44, 54)’ represent median (IQR). Statistical comparisons were performed using Pearson chi-squared test or Mann-Whitney U test as appropriate. Abbreviations: a –  Mann-Whitney test; CAD – Coronary Artery Disease; PAD – Peripheral Arterial Disease; DM – Diabetes Mellitus; CVS – Cardiovascular Disease; MI – Myocardial Infarction; PCI – Percutaneous Coronary Intervention; CABG – Coronary Artery Bypass Graft; n – number

Variable	Non-premature CAD (N=1,363)	Premature CAD (N=637)	P value
Age (years)	71 (65, 79)	49 (44, 54)	<.001^a^
Male sex	1092 (80.1%)	436 (68.4%)	< .001>
Smoking	128 (9.4%)	209 (32.8%)	< .001>
Family history of dyslipidemia	50 (3.7%)	130 (20.4%)	< .001>
DM	994 (72.9%)	336 (52.7%)	< .001>
Hypertension	1036 (76.0%)	342 (53.7%)	< .001>
Dyslipidemia	754 (55.3%)	324 (50.9%)	.063
PVD	38 (2.8%)	8 (1.3%)	.033
CVS	100 (7.3%)	35 (5.5%)	.126
Prior MI	224 (16.4%)	74 (11.6%)	.005
Prior angina	395 (29.0%)	124 (19.5%)	< .001>
Prior PCI	327 (24.0%)	110 (17.3%)	.001
Prior CABG	96 (7.0%)	19 (3.0%)	< .001>
Repeat attack	433 (31.8%)	139 (21.8%)	< .001>
Prior statin intake	844 (61.9%)	312 (49.0%)	< .001>

Lipid parameters were significantly elevated in the premature CAD group, with serum total cholesterol (174.0 mg/dl vs. 145.0 mg/dl, p<0.001), LDL-C (108.3 mg/dl vs. 85.1 mg/dl, p<0.001) and corrected LDL-C (155.8 mg/dl vs 147.3 mg/dl, p<0.001). A greater proportion of premature CAD patients had LDL-C ≥100 mg/dl (57.1% vs. 38.8%, p < .001) and cholesterol ≥200 mg/dl (28.4% vs 15%, p<0.001).

**Figure 1:**
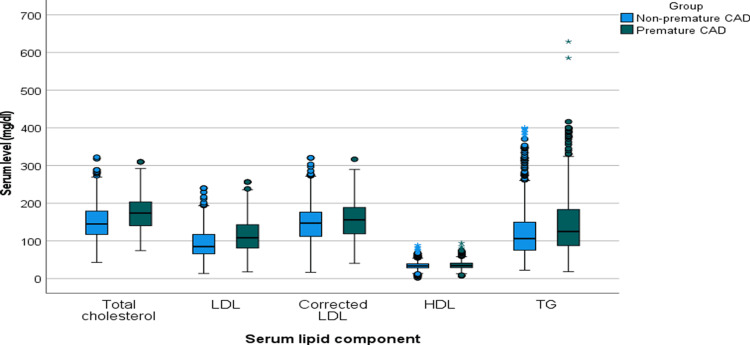
Box plot illustrating the serum lipid levels in patients with premature and non-premature coronary artery disease (CAD). The Y-axis indicates the percentage of patients. The box represents the interquartile range, with the median indicated by the central line. Whiskers show minimum and maximum values excluding outliers (round markers) and extreme values (asterisks). Abbreviations: LDL – Low-Density Lipoprotein; TG – Triglyceride

Triglyceride levels were also higher (median 124.9 mg/dl vs. 106.3 mg/dl; p<0.001), with more patients exceeding 150 mg/dl (37.0% vs. 24.9%, p<0.001). HDL-C was marginally higher in premature CAD (p=0.004), as shown in Table 2 and Figure 1.

**Table 2:** Laboratory findings and clinical scores for familial hypercholesterolemia. Values are shown as median (IQR) or n (%). ‘Corrected LDL’ refers to LDL adjusted for statin use. Statistical tests: a = Mann-Whitney U test; b = Linear-by-linear association. Abbreviations: CAD – Coronary Artery Disease; LDL-C – Low-Density Lipoprotein Cholesterol; HDL-C – High-Density Lipoprotein Cholesterol; TG – Triglyceride; FH – Familial Hypercholesterolemia

Variable	Non-premature CAD (N=1,363)	Premature CAD (N=637)	P value
Cholesterol (mg/dl)	145.0 (117.2, 179.4)	174.0 (140.4, 203.4)	<.001^a^
Cholesterol ≥200 mg/dl	204 (15.0%)	181 (28.4%)	< .001>
LDL (mg/dl)	85.1 (65.7, 117.20	108.3 (81.2, 143.1)	<.00^ a^
LDL ≥100 mg/dl	529 (38.8%)	364 (57.1%)	< .001>
Corrected LDL (mg/dl)	147.3 (112.1, 176.3)	155.8 (118.7, 188.7)	<.001^a^
Corrected LDL ≥100 mg/dl	1096 (80.4%)	533 (83.7%)	.080
HDL (mg/dl)	33.6 (28.6, 39.4)	34.4 (29.0, 41.0)	.004^a^
HDL <60 mg/dl	1342 (98.5%)	614 (96.4%)	.003
TG (mg/dl)	106.3 (75.3, 149.7)	124.9 (87.7, 183.3)	<.001^a^
TG ≥150 mg/dl	340 (24.9%)	236 (37.0%)	< .001>
Dutch Lipid Clinic Network Criteria for FH			<.001^b^
Unlikely	1094 (80.3%)	375 (58.9%)	
Possible	249 (18.3%)	185 (29.0%)	
Probable/Definite	20 (1.5%)	77 (12.1%)	
Simon Broome Diagnostic Criteria for FH			<.001^b^
Unlikely	1322 (97.0%)	446 (70.0%)	
Possible	41 (3.0%)	188 (29.5%)	
Definite	0 (0.0%)	3 (0.5%)	

**Figure 2:**
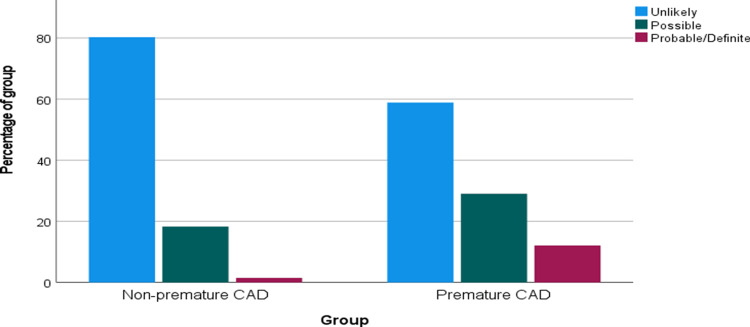
Bar chart showing the distribution of Dutch Lipid Clinic Network criteria classifications in patients with premature and non-premature coronary artery disease (CAD).

**Figure 3:**
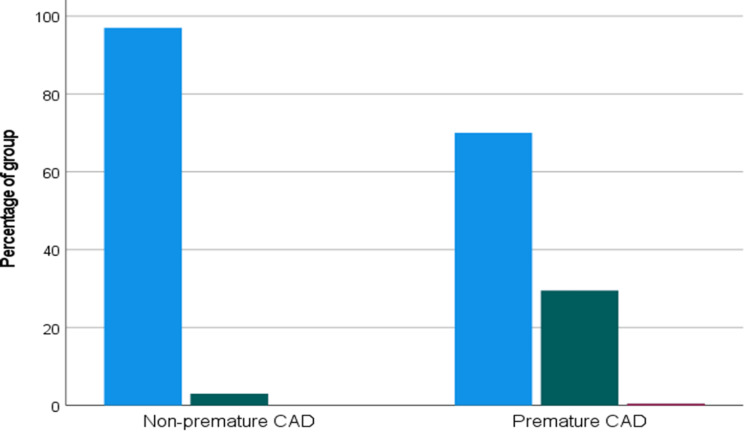
Bar chart showing the Simon Broome Diagnostic Criteria categories in patients with premature and non-premature coronary artery disease (CAD). The Y-axis represents the percentage of patients.

FH risk scores were significantly higher among patients with premature CAD. According to the DLCN criteria, possible FH was observed in 185/637 (29.0%) patients with premature CAD compared with 249/1363 (18.3%) in the non-premature group, while probable/definite FH occurred in 77/637 (12.1%) versus 20/1363 (1.5%), respectively (both p<0.001). Similarly, the Simon Broome diagnostic criteria demonstrated higher rates of possible FH in the premature CAD group (188/637 [29.5%] vs. 41/1363 [3.0%]) and definite FH (3/637 [0.5%] vs. 0/1363 [0.0%]; p<0.001), as shown in Table 2 and Figures 2, 3.

Regarding lipid-lowering therapy, prior statin use was significantly lower in the premature CAD group compared to the non-premature group (49.0% vs. 61.9%; p<0.001), as shown in Table 1. During hospitalization, nearly all patients received statins in accordance with guideline-recommended care for ACS. At discharge, statin prescription rates remained high and comparable between groups, while ezetimibe (10.4% vs. 6.2%) and evolocumab (1.9% vs. 0.7%) use was higher in the premature CAD group, reflecting a trend toward more intensive lipid-lowering in younger high-risk patients. These patterns are detailed in Table 3. Also, coronary angiography was performed more often in premature CAD (90.7% vs. 83.6%, p<0.001), as shown in Table 3.

**Table 3:** Cardiological diagnosis and in-hospital management. Data are shown as n (%) or median (IQR). Statistical tests: a = Linear-by-linear association; b = Fisher’s exact test; c = Fisher-Freeman-Halton’s exact test. Abbreviations: CAD – Coronary Artery Disease; UA – Unstable Angina; STEMI – ST-Elevation Myocardial Infarction; NSTEMI – Non-STEMI; ECG – Electrocardiogram; MVD – Multi-Vessel Disease; LM – Left Main; LAD – Left Anterior Descending; LCX – Left Circumflex; OM – Obtuse Marginal; RCA – Right Coronary Artery; LIMA – Left Internal Mammary Artery; OAC – Oral Anticoagulant; NOAC – Novel Oral Anticoagulant; CABG – Coronary Artery Bypass Graft

Variable	Non-premature CAD (N=1,363)	Premature CAD (N=637)	P value
Diagnosis			.622^a^
Unstable angina	339 (24.9%)	182 (28.6%)	
NSTEMI	668 (49.0%)	254 (39.9%)	
STEMI	356 (26.1%)	201 (31.6%)	
In-hospital medication			
Aspirin	1363 (100.0%)	637 (100.0%)	NA
P2Y12 inhibitor	1363 (100.0%)	637 (100.0%)	NA
Statin	1363 (100.0%)	634 (99.5%)	.032^b^
Antihypertensive	1070 (78.5%)	396 (62.2%)	< .001>
Coronary angiography done	1140 (83.6%)	578 (90.7%)	< .001>
Management			.061
Conservative	352 (25.8%)	190 (29.8%)	
Interventional	1011 (74.2%)	447 (70.2%)	
Reason for conservative treatment			<.001^c^
No coronary angiography	223/352 (63.4%)	59/190 (31.1%)	
Normal coronary angiography	45/352 (12.8%)	71/190 (37.4%)	
Insignificant atherosclerotic CAD	72/352 (20.5%)	40/190 (21.1%)	
MVD	5/352 (1.4%)	3/190 (1.6%)	
Slow flow	7/352 (2.0%)	17/190 (8.9%)	
Interventions			
Stent implantation	849 (62.3%)	390 (61.2%)	.648
Balloon dilatation	16 (1.2%)	3 (0.5%)	.131
CABG	153 (11.2%)	56 (8.8%)	.097
Medications on discharge			
Aspirin	1322 (97.0%)	606 (95.1%)	.038
P2Y12 inhibitor	1311 (96.2%)	557 (87.4%)	< .001>
Statin	1359 (99.7%)	629 (98.7%)	.024^ b^
High-dose statin	1284 (94.2%)	584 (91.7%)	.042
Ezetimibe	84 (6.2%)	66 (10.4%)	.001
Evolocumab	10 (0.7%)	12 (1.9%)	.022
Ezetimibe + Evolocumab	6 (0.4%)	3 (0.5%)	>.999^ b^
OAC	29 (2.1%)	22 (3.5%)	.080
NOAC	140 (10.3%)	27 (4.2%)	< .001>
Antihypertensive	1181 (86.6%)	447 (70.2%)	< .001>
Variable	Non-premature CAD (N=1,363)	Premature CAD (N=637)	P value

**Figure 4:**
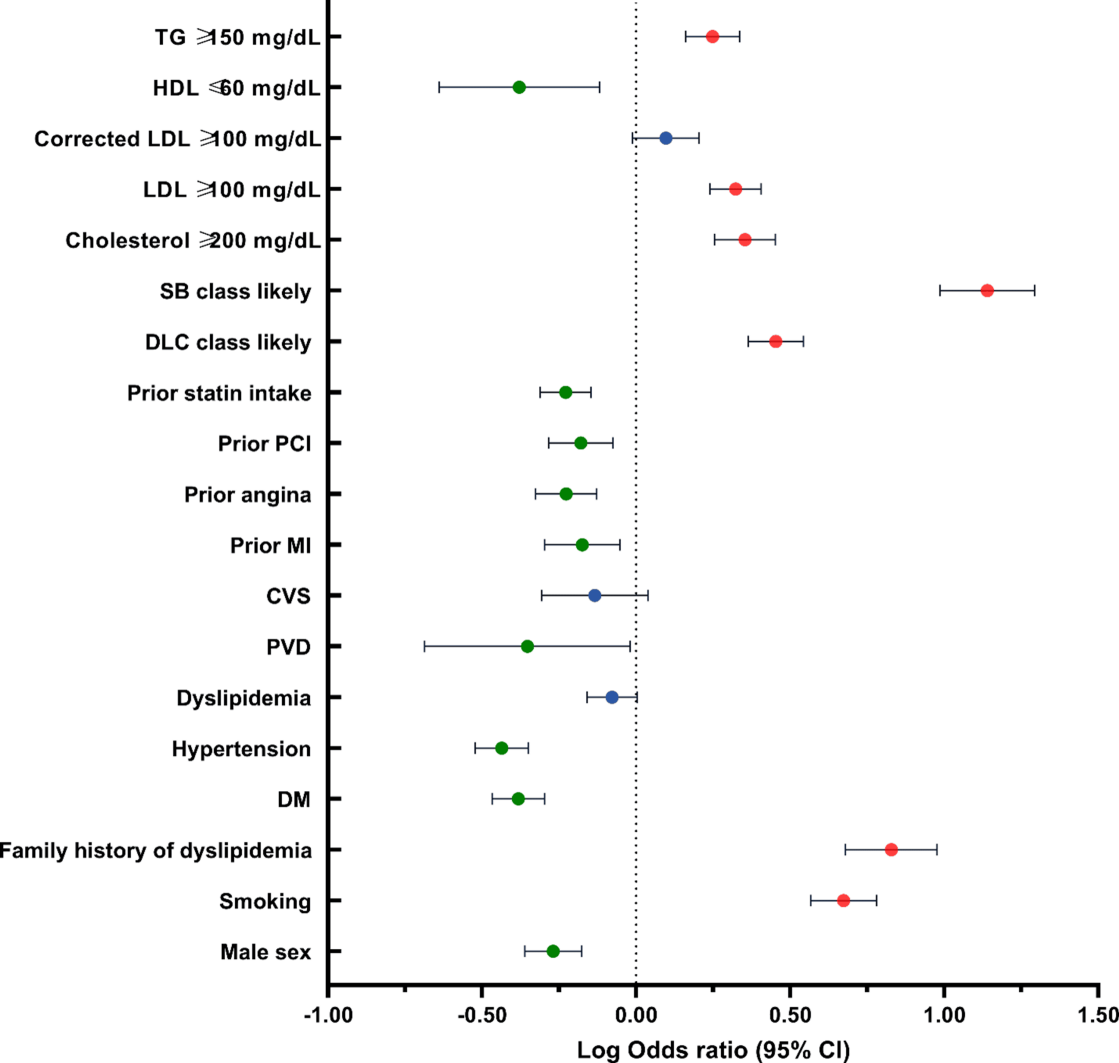
Forest plot of log-transformed odds ratios of risk factors for premature coronary artery disease (CAD). Red markers indicate statistically significant increased risk, green markers indicate statistically significant decreased risk, and blue markers indicate non-significant associations. Abbreviations: DM – Diabetes Mellitus; PAD – Peripheral Arterial Disease; CVS – Cardiovascular Disease; MI – Myocardial Infarction; PCI – Percutaneous Coronary Intervention; PVD – Peripheral Vascular Disease; LDL-C – Low-Density Lipoprotein Cholesterol; HDL-C – High-Density Lipoprotein Cholesterol; TG – Triglyceride; DLC  –Dutch Lipid Clinic; SB – Simon Broome.

Multivariate risk analysis revealed several significant positive predictors for premature CAD: smoking (OR=4.71, 95% CI: 3.68-6.03, p<0.0001), family history of dyslipidemia (OR=6.73, p<0.0001), LDL-C ≥100 mg/dl (OR=2.10, p<0.0001), total cholesterol ≥200 mg/dl (OR=2.26, p<0.0001), and TG ≥150 mg/dl (OR 1.77, 95% CI 1.45-2.17). Negative predictors were diabetes mellitus (OR=0.41, p<0.0001), hypertension (OR=0.37, p<0.0001), and prior statin use (OR=0.59, p<0.0001) as presented in Table 4 and Figure 4.

**Table 4:** Multivariate logistic regression analysis for predictors of premature coronary artery disease (CAD). Values include odds ratios (OR), 95% confidence intervals (CI), z-statistic, and p-value. Statistical significance assessed with the Z-test. Abbreviations: DM – Diabetes Mellitus; PVD – Peripheral Vascular Disease; CVD – Cardiovascular Disease; MI – Myocardial Infarction; PCI – Percutaneous Coronary Intervention; LDL-C – Low-Density Lipoprotein Cholesterol; HDL-C – High-Density Lipoprotein Cholesterol; TG – Triglyceride.

Variable	Odds ratio	Lower 95% CI	Upper 95% CI	z-statistic	P value
Male sex	0.54	0.43	0.67	5.683	< .0001>
Smoking	4.71	3.68	6.03	12.353	< .0001>
Family history of dyslipidemia	6.73	4.78	9.48	10.933	< .0001>
DM	0.41	0.34	0.50	8.803	< .0001>
Hypertension	0.37	0.30	0.45	9.888	< .0001>
Dyslipidemia	0.84	0.69	1.01	1.862	.0627
PVD	0.44	0.21	0.96	2.074	.0381
CVD	0.73	0.49	1.09	1.525	.1273
Prior MI	0.67	0.50	0.89	2.805	.005
Prior angina	0.59	0.47	0.74	4.494	< .0001>
Prior PCI	0.66	0.52	0.84	3.375	.0007
Prior statin intake	0.59	0.49	0.71	5.438	< .0001>
Cholesterol ≥200 mg/dl	2.26	1.80	2.83	7.003	< .0001>
LDL ≥100 mg/dl	2.10	1.74	2.54	7.622	< .0001>
Corrected LDL ≥100 mg/dl	1.25	0.97	1.60	1.747	.081
HDL ≤60 mg/dl	0.42	0.23	0.76	2.855	.004
TG ≥150 mg/dl	1.77	1.45	2.17	5.537	< .0001>

## Discussion

This study provides a comprehensive comparison between premature and non-premature CAD in patients presenting with ACS, revealing clear differences in demographics, risk factors, lipid abnormalities, diagnostic assessment, and in-hospital management. Patients with premature CAD were younger and predominantly male, consistent with prior literature [16,17]. Smoking (OR: 4.71) and family history of dyslipidemia (OR: 6.73) emerged as the strongest factors associated with premature CAD, in agreement with large international studies such as INTERHEART that identified smoking and dyslipidemia as major drivers of early myocardial infarction [18,19].

Unlike older patients, in whom metabolic syndromes are dominant contributors, premature CAD appears more strongly linked to behavioral and genetic risk factors. The lower prevalence of diabetes and hypertension in the premature group (p<0.001 for both) supports a distinct pathogenic pathway and is consistent with regional data showing a reduced metabolic burden among younger patients despite serious cardiovascular events [20].

Atherogenic lipid abnormalities were a defining feature of premature CAD in our cohort. Patients with early-onset disease demonstrated significantly higher total cholesterol, LDL-C, corrected LDL-C, and triglyceride levels (all p<0.001). Elevated lipid thresholds, including total cholesterol ≥200 mg/dL, LDL-C ≥100 mg/dL, and triglycerides ≥150 mg/dL, were independently associated with premature CAD (OR: 2.10 and OR: 1.77), underscoring their role in early atherosclerosis development [21].

Clinical assessment using FH diagnostic tools further highlighted the genetic contribution to premature disease. The DLCN criteria identified higher rates of both possible and probable/definite FH in premature CAD, and similar findings were observed with the Simon Broome classification. These results are consistent with prior evidence demonstrating underdiagnosis and undertreatment of FH, particularly in younger adults presenting with ACS [22,23]. Given that untreated FH can increase the risk of premature CAD up to 20-fold [24], early detection and family-based cascade screening are essential, especially in regions with limited access to specialized lipid services. Despite differing risk profiles, both groups presented with similar ACS subtypes, suggesting convergence of ischemic outcomes despite distinct etiologies. Premature patients were more likely to undergo coronary angiography and receive interventional therapy, potentially reflecting clinical decision-making patterns and selection bias. Notably, a substantial proportion of conservatively managed premature patients had non-obstructive coronary findings, suggesting mechanisms such as microvascular dysfunction or vasospasm that are increasingly recognized in younger populations [25].

Discharge pharmacotherapy was broadly similar between groups, with high statin and aspirin use. However, younger patients more frequently received ezetimibe and PCSK9 inhibitors, consistent with guideline-directed management of FH or persistent hyperlipidemia [26]. Although corrected LDL-C was slightly higher in premature CAD, it was not significant in multivariate analysis (p=0.081), possibly reflecting acute lipid variability and methodological limitations [27].

The inverse association of prior statin therapy, diabetes, and hypertension with premature CAD may reflect delayed disease onset in treated chronic conditions, survivorship effects, and the stronger influence of genetic and behavioral factors, particularly smoking in younger individuals. The widespread use of intensive lipid-lowering therapy in premature CAD aligns with contemporary guideline recommendations for aggressive risk reduction in high-risk young patients [28].

These findings underscore the need for targeted public health strategies focusing on early identification of at-risk young individuals, particularly smokers and those with a family history of dyslipidemia. Recommended approaches include early lipid screening, age-adjusted risk assessment tools, and implementation of cascade testing and universal cholesterol screening in young adults, as endorsed by international guidelines [29,30].

Social determinants of health also play a critical role in premature CAD. Socioeconomic status, education, occupational stress, healthcare access, and urban lifestyle influence both disease prevalence and severity. Individuals from disadvantaged backgrounds are more likely to smoke, maintain unhealthy diets, and receive inadequate preventive care, all of which accelerate atherosclerosis [31].

Addressing these upstream determinants through public health policy and education is essential to reduce disparities in early cardiovascular disease.

Limitations

This study has several limitations. It’s a cross-sectional observational study that prevents the assessment of causality or long-term outcomes. The absence of genetic confirmation for FH limits diagnostic precision. Additionally, the single-center setting may reduce the generalizability of our findings. Lifestyle, dietary patterns, and BMI were not evaluated, and the imaging data lacked advanced diagnostic modalities. Future multicenter, prospective studies incorporating genetics are needed.

## Conclusions

Premature CAD in patients presenting with ACS shows a distinct profile with higher rates of smoking, dyslipidemia, and FH, but fewer traditional comorbidities. This study is unique in applying dual FH diagnostic criteria in a large, real-world cohort from an underrepresented region. These findings highlight the need for early detection and aggressive lipid-lowering strategies in high-risk younger adults.

## References

[1] Jafar TH, Jafary FH, Jessani S, Chaturvedi N (2005). Heart disease epidemic in Pakistan: women and men at equal risk. Am Heart J.

[2] Nordestgaard BG, Chapman MJ, Humphries SE (2013). Familial hypercholesterolaemia is underdiagnosed and undertreated in the general population: guidance for clinicians to prevent coronary heart disease: consensus statement of the European Atherosclerosis Society. Eur Heart J.

[3] Cuchel M, Bruckert E, Ginsberg HN (2014). Homozygous familial hypercholesterolaemia: new insights and guidance for clinicians to improve detection and clinical management. A position paper from the Consensus Panel on Familial Hypercholesterolaemia of the European Atherosclerosis Society. Eur Heart J.

[4] Benn M, Watts GF, Tybjærg-Hansen A, Nordestgaard BG (2016). Mutations causative of familial hypercholesterolaemia: screening of 98 098 individuals from the Copenhagen General Population Study estimated a prevalence of 1 in 217. Eur Heart J.

[5] Santos RD, Gidding SS, Hegele RA (2016). Defining severe familial hypercholesterolaemia and the implications for clinical management: a consensus statement from the International Atherosclerosis Society Severe Familial Hypercholesterolemia Panel. Lancet Diabetes Endocrinol.

[6] Abul-Husn NS, Manickam K, Jones LK (2016). Genetic identification of familial hypercholesterolemia within a single U.S. health care system. Science.

[7] Stringhini S, Sabia S, Shipley M, Brunner E, Nabi H, Kivimaki M, Singh-Manoux A (2010). Association of socioeconomic position with health behaviors and mortality. JAMA.

[8] Thygesen K, Alpert JS, Jaffe AS, Chaitman BR, Bax JJ, Morrow DA, White HD (2018). Fourth universal definition of myocardial infarction (2018). Circulation.

[9] Ibanez B, James S, Agewall S (2018). 2017 ESC guidelines for the management of acute myocardial infarction in patients presenting with ST-segment elevation: The Task Force for the management of acute myocardial infarction in patients presenting with ST-segment elevation of the European Society of Cardiology (ESC). Eur Heart J.

[10] Braga F, Pasqualetti S, Borrillo F (2023). Definition and application of performance specifications for measurement uncertainty of 23 common laboratory tests: linking theory to daily practice. Clin Chem Lab Med.

[11] Law MR, Wald NJ, Rudnicka AR (2003). Quantifying effect of statins on low density lipoprotein cholesterol, ischaemic heart disease, and stroke: systematic review and meta-analysis. BMJ.

[12] Davidson MH, Ballantyne CM, Kerzner B (2004). Efficacy and safety of ezetimibe coadministered with statins: randomised, placebo-controlled, blinded experience in 2382 patients with primary hypercholesterolemia. Int J Clin Pract.

[13] Bruckert E, De Gennes JL, Malbecq W, Baigts F (1995). Comparison of the efficacy of simvastatin and standard fibrate therapy in the treatment of primary hypercholesterolemia and combined hyperlipidemia. Clin Cardiol.

[14] Ballantyne C, Gleim G, Liu N, Sisk CM, Johnson-Levonas AO, Mitchel Y (2012). Effects of coadministered extended-release niacin/laropiprant and simvastatin on lipoprotein subclasses in patients with dyslipidemia. J Clin Lipidol.

[15] Eriksson M, Hådell K, Holme I (1998). Compliance and efficacy of pravastatin and cholestyramine treatment. J Intern Med.

[16] Arora S, Stouffer GA, Kucharska-Newton AM (2019). Twenty year trends and sex differences in young adults hospitalized with acute myocardial infarction. Circulation.

[17] Maas AH, Appelman YE (2010). Gender differences in coronary heart disease. Neth Heart J.

[18] Yusuf S, Hawken S, Ounpuu S (2004). Effect of potentially modifiable risk factors associated with myocardial infarction in 52 countries (INTERHEART). Lancet.

[19] D’Agostino RB, Vasan RS, Pencina MJ (2008). General cardiovascular risk profile for use in primary care. Circulation.

[20] Singh A, Collins BL, Gupta A (2018). Cardiovascular risk and statin eligibility of young adults after myocardial infarction: the YOUNG-MI registry. J Am Coll Cardiol.

[21] Mach F, Baigent C, Catapano AL (2020). 2019 ESC/EAS Guidelines for the management of dyslipidaemias: lipid modification to reduce cardiovascular risk. Eur Heart J.

[22] Benn M, Watts GF, Tybjaerg-Hansen A, Nordestgaard BG (2012). Familial hypercholesterolemia in the Danish general population: prevalence, coronary artery disease, and cholesterol-lowering medication. J Clin Endocrinol Metab.

[23] Khera AV, Won HH, Peloso GM (2016). Diagnostic yield and clinical utility of sequencing familial hypercholesterolemia genes. J Am Coll Cardiol.

[24] Goldberg AC, Hopkins PN, Toth PP (2011). Familial hypercholesterolemia: screening, diagnosis and management of pediatric and adult patients: clinical guidance from the National Lipid Association Expert Panel on Familial Hypercholesterolemia. J Clin Lipidol.

[25] Knuuti J, Wijns W, Saraste A (2020). 2019 ESC Guidelines for the diagnosis and management of chronic coronary syndromes. Eur Heart J.

[26] Grundy SM, Stone NJ, Bailey AL (2019). 2018 AHA/ACC/AACVPR/AAPA/ABC/ACPM/ADA/AGS/APhA/ASPC/NLA/PCNA multisociety guideline on the management of blood cholesterol: a report of the American College of Cardiology/American Heart Association Task Force on Clinical Practice Guidelines. Circulation.

[27] Tyurina AV, Kukharchuk VV, Soloveva EY (2022). Association of various lipid parameters with premature coronary artery disease in men. Russ J Cardiol.

[28] Ray KK, Bays HE, Catapano AL (2019). Safety and efficacy of bempedoic acid to reduce LDL cholesterol. N Engl J Med.

[29] Gidding SS, Champagne MA, de Ferranti SD (2015). The agenda for familial hypercholesterolemia: a scientific statement from the American Heart Association. Circulation.

[30] Wiegman A, Gidding SS, Watts GF (2015). Familial hypercholesterolaemia in children and adolescents: gaining decades of life by optimizing detection and treatment. Eur Heart J.

[31] Havranek EP, Mujahid MS, Barr DA (2015). Social determinants of risk and outcomes for cardiovascular disease: a scientific statement from the American Heart Association. Circulation.

